# TLR4 as a Potential Target of Me-PFOSA-AcOH Leading to Cardiovascular Diseases: Evidence from NHANES 2013–2018 and Molecular Docking

**DOI:** 10.3390/toxics12100693

**Published:** 2024-09-25

**Authors:** Zhilei Mao, Yanling Chen, Haixin Li, Qun Lu, Kun Zhou

**Affiliations:** 1 Changzhou Maternity and Child Health Care Hospital, Changzhou Medical Center, Nanjing Medical University, Changzhou 213003, China; mao598808386@126.com (Z.M.); sungirllhx@163.com (H.L.); 2 State Key Laboratory of Reproductive Medicine and Offspring Health, Center for Global Health, School of Public Health, Nanjing Medical University, Nanjing 211166, China; cyanling9211@163.com; 3 Key Laboratory of Modern Toxicology of Ministry of Education, School of Public Health, Nanjing Medical University, Nanjing 211166, China; 4 Department of Prenatal Diagnosis, Women’s Hospital of Nanjing Medical University, Nanjing 210004, China; 5 Department of Epidemiology, Center for Global Health, School of Public Health, Nanjing Medical University, Nanjing 211166, China

**Keywords:** perfluoroalkyl substances, cardiovascular diseases, Me-PFOSA-AcOH, NHANES, PLS-DA, molecular docking

## Abstract

Background: Concerns have been raised regarding the effects of perfluoroalkyl substance (PFAS) exposure on cardiovascular diseases (CVD), but clear evidence linking PFAS exposure to CVD is lacking, and the mechanism remains unclear. Objectives: To study the association between PFASs and CVD in U.S. population, and to reveal the mechanism of PFASs’ effects on CVD. Methods: To assess the relationships between individual blood serum PFAS levels and the risk of total CVD or its subtypes, multivariable logistic regression analysis and partial least squares discriminant analysis (PLS-DA) were conducted on all participants or subgroups among 3391 adults from the National Health and Nutrition Examination Survey (NHANES). The SuperPred and GeneCards databases were utilized to identify potential targets related to PFAS and CVD, respectively. Gene ontology (GO) and Kyoto Encyclopedia of Genes and Genomes (KEGG) enrichment analyses of intersection genes were performed using Metascape. Protein interaction networks were generated, and core targets were identified with STRING. Molecular docking was achieved using Autodock Vina 1.1.2. Results: There was a positive association between Me-PFOSA-AcOH and CVD (OR = 1.28, *p* = 0.022), especially coronary heart disease (CHD) (OR = 1.47, *p* = 0.007) and heart attack (OR = 1.58, *p* < 0.001) after adjusting for all potential covariates. Me-PFOSA-AcOH contributed the most to distinguishing between individuals in terms of CVD and non-CVD. Significant moderating effects for Me-PFOSA-AcOH were observed in the subgroup analysis stratified by sex, ethnicity, education level, PIR, BMI, smoking status, physical activity, and hypertension (*p* < 0.05). The potential intersection targets were mainly enriched in CVD-related pathways, including the inflammatory response, neuroactive ligand–receptor interaction, MAPK signaling pathway, and arachidonic acid metabolism. TLR4 was identified as the core target for the effects of Me-PFOSA-AcOH on CVD. Molecular docking results revealed that the binding energy of Me-PFOSA-AcOH to the TLR4-MD-2 complex was −7.2 kcal/mol, suggesting that Me-PFOSA-AcOH binds well to the TLR4-MD-2 complex. Conclusions: Me-PFOSA-AcOH exposure was significantly associated with CVD. Network toxicology and molecular docking uncovered novel molecular targets, such as TLR4, and identified the inflammatory and metabolic mechanisms underlying Me-PFOSA-AcOH-induced CVD.

## 1. Introduction

PFASs are recognized as persistent environmental contaminants, exhibiting chemical, thermal, and biological stability [1]. PFASs are a group of substances commonly found in everyday consumer items, including food packaging, non-stick kitchenware, and water-resistant coatings [2]. As pervasive environmental contaminants, PFASs pose significant challenges due to their widespread distribution [3], persistence [4], and potential health risks [5]. PFASs can infiltrate the human body through different exposure pathways, such as contamination of food and water, inhalation, and contact with the skin [6]. Previous studies in humans and animals showed that PFAS exposure led to various health issues, such as cancers [7], obesity [8], adverse pregnancy outcomes [9], abnormal glucose and lipid metabolism [10], and CVD [11]. 

CVD includes a variety of disorders impacting the heart and blood vessels, such as coronary artery disease, heart failure, stroke, angina, and peripheral vascular disease [12]. CVD is a major worldwide public health issue and has emerged as a leading cause of illness and death among both men and women in the United States and globally [13]. The morbidity of CVD in the US represents an obvious health burden with far-reaching implications for individuals, communities, and the healthcare system [14]. Although conventional risk factors, like smoking, hypertension, diabetes, and abnormal lipid levels, have been thoroughly examined and identified as contributors to CVD [15], emerging study suggests that environmental contaminants may also play an important role in the pathogenesis of CVD [16]. Urinary BPA was linked to a higher risk of CVD in a population study [17]. Di(2-ethylhexyl) phthalate (DEHP) exposure has been reported to elevate CVD risk in animals [18]. Exposure to a mixture of heavy metals has been associated with an increased risk of CVD [19]. 

Previously, a minority of studies with limitations have reported a connection between PFAS exposure and CVD [20,21]. In a cohort study, the relationship was investigated exclusively for perfluorohexanesulfonic acid (PFHxS), perfluorooctanoic acid (PFOA), and perfluorooctane sulfonic acid (PFOS) with just three types of cardiovascular disease (myocardial infarction, ischemic stroke, or heart failure) [22]. Another study was conducted to examine the association between exposure to PFASs and the risk of acute coronary syndrome, but it involved only a relatively small sample size [23]. More importantly, inconsistent findings were usually observed in several previous studies [24,25,26]. Results from two population-based cohort studies in Sweden did not show an increased risk of CVD with moderately elevated PFAS levels. However, a meta-analysis of five separate cohort studies suggested a slight negative association between PFOA levels and CVD, further indicating that higher PFAS levels are not connected to a greater risk of CVD [22]. Thus, the association between PFAS exposure and cardiovascular disease remains insufficiently clarified, and the mechanisms by which PFAS exposure causes CVD are still unclear. 

The NHANES is an extensive, ongoing survey carried out by the Centers for Disease Control and Prevention (CDC) that gathers data on the health and nutritional status of the U.S. population [27]. NHANES offers a significant resource for examining the link between environmental exposures, like PFASs, and different health outcomes [28,29]. In this study, a cross-sectional design was used to investigate the association between blood serum PFAS levels and the risk of total CVD or its subtypes by analyzing data from NHANES 2013–2018. To assess the relationship between PFAS exposure and CVD risk, multivariable logistic regression models and a PLS-DA model were employed. Additionally, network toxicology and molecular docking were utilized to explore the mechanisms of PFAS activity within the context of CVD [30]. 

## 2. Results

### 2.1. Baseline Characteristics

In the present study, the demographic characteristics of the study were shown in Table 1, comprising a total of 3391 individuals. Among them, 371 participants were in the CVD group, while 3020 were in the non-CVD group. No significant difference was observed in sex ratio between these two groups. Individuals with CVD, in comparison to those without, were characterized by advanced age, a higher likelihood of being non-Hispanic, being smokers, and having BMI above the normal range, performing no physical activity, and having a family history of CVD and hypertension. 

### 2.2. Serum Levels of PFASs 

Next, we conducted an analysis of blood serum levels of PFASs in the adult participants. As presented in Table 2, the levels of PFHxS, PFNA, n-PFOS, Sm-PFOS, and Me-PFOSA-AcOH were significantly increased in the CVD group. These results indicate that exposure to these PFASs might be associated with CVD. 

### 2.3. Association between PFASs and CVD 

**Figure 1:**
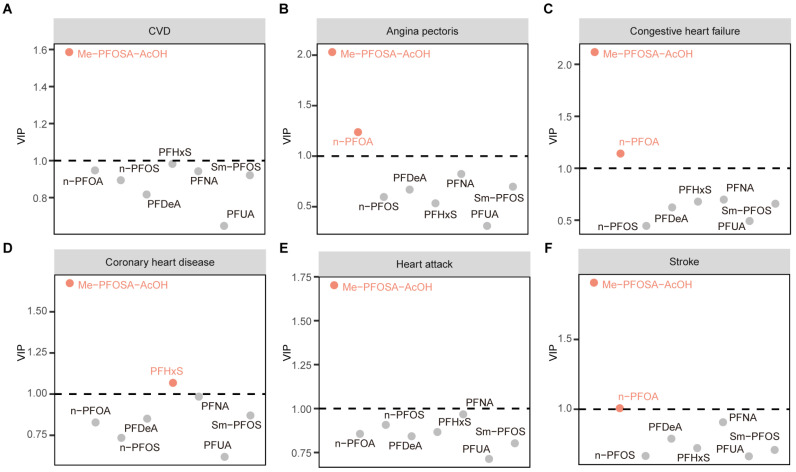
VIP plot of the PLS-DA model for (**A**) the total CVD; (**B**) angina pectoris; (**C**) congestive heart failure; (**D**) coronary heart disease; (**E**) heart attack and (**F**) stroke.

Next, we performed multivariable logistic regression analysis to examine the association between PFASs and CVD, including its subtypes. In Table 3, it is indicated that after fully adjusting for potential covariates in Model 3, significant relationships with CVD were observed for PFUA, n-PFOA, and Me-PFOSA-AcOH (OR = 1.32, 95% CI: 1.08, 1.61; OR = 0.73, 95% CI: 0.56, 0.96; OR = 1.28, 95% CI: 1.05, 1.56, respectively). We further explored the association between PFASs and CVD subtypes. As demonstrated in Table S1, following adjustments for all covariates, we observed that Me-PFOSA-AcOH was associated with coronary heart disease (OR = 1.47, 95% CI: 1.13, 1.92) and heart attack (OR = 1.58, 95% CI: 1.26, 1.99). To further identify which type of PFASs contribute the most to distinguishing between CVD and non-CVD cases, we employed a PLS-DA model. Discriminant variables were identified through the use of variable importance in projection (VIP) scores, offering an overall assessment of how each variable affects the PLS components. Variables with a VIP score higher than 1.0 were considered to be discriminant [31]. As depicted in Figure 1A, Me-PFOSA-AcOH contributed the most to distinguishing between individuals with CVD and those without CVD, with a VIP score > 1.5. Additionally, we observed that Me-PFOSA-AcOH also played a significant role in distinguishing participants with CVD subtypes from those without CVD subtypes (Figure 1B–F). Overall, these results confirm that Me-PFOSA-AcOH was strongly associated with CVD, particularly coronary heart disease and heart attack. 

### 2.4. Subgroup Analysis

**Figure 2:**
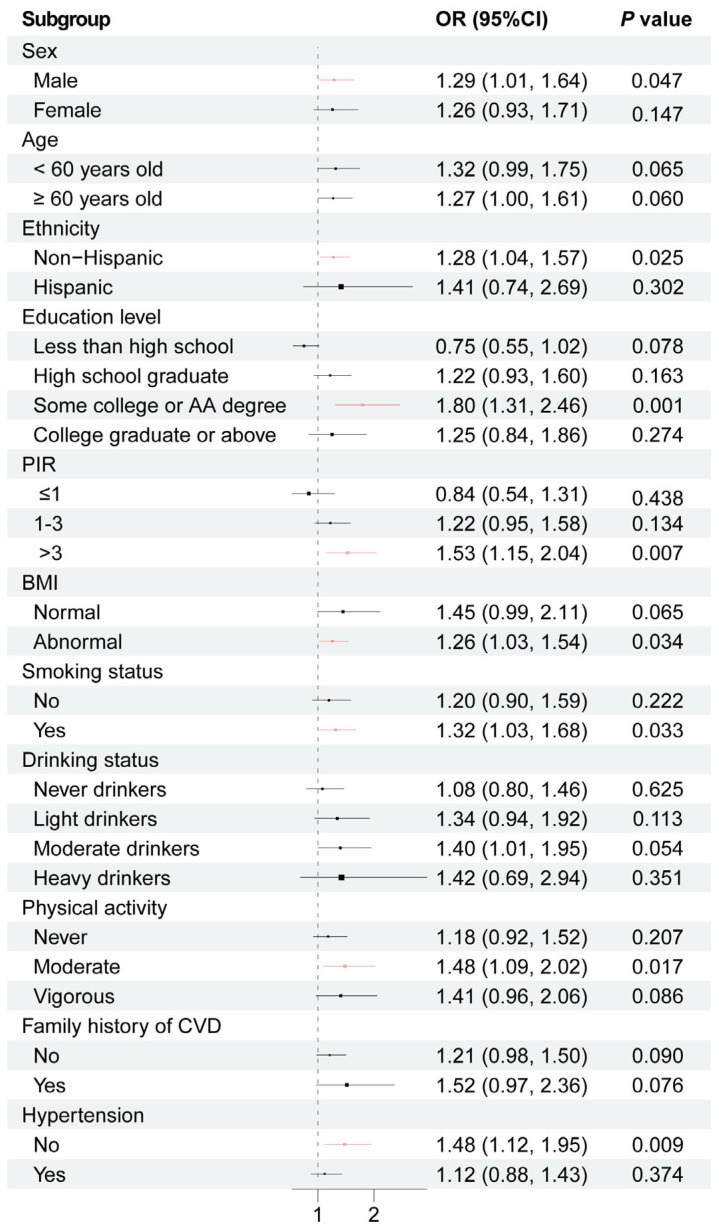
Subgroup analysis for the relationship between Me-PFOSA-AcOH and CVD, adjusted for sex, age, ethnicity, education level, PIR, physical activity, smoking status, drinking status, family history of CVD, BMI, and hypertension, with the exception of the subgroup variable.

To evaluate the stability of the association between Me-PFOSA-AcOH and CVD across various subgroups, we performed a subgroup analysis. As shown in Figure 2, for the subgroups stratified by sex, ethnicity, education level, PIR, BMI, smoking status, physical activity, hypertension, a significant relationship of Me-PFOSA-AcOH with CVD was detected in each subgroup (*p* < 0.05). In detail, a statistically significant positive association between Me-PFOSA-AcOH and CVD was observed in the following subgroups: males (OR = 1.29, 95% CI: 1.01, 1.64, *p* = 0.047), non-Hispanic individuals (OR = 1.28, 95% CI: 1.04, 1.57, *p* = 0.025), those with some college or AA degree (OR = 1.80, 95% CI: 1.31, 2.46, *p* = 0.001), individuals with a PIR > 3 (OR = 1.53, 95% CI: 1.15, 2.04, *p* = 0.007), those with abnormal BMI (OR = 1.26, 95% CI: 1.03, 1.54, *p* = 0.034), smokers (OR = 1.32, 95% CI: 1.03, 1.68, *p* = 0.033), individuals with moderate physical activity (OR = 1.48, 95% CI: 1.09, 2.02, *p* = 0.017), or those without hypertension (OR = 1.48, 95% CI: 1.12, 1.95, *p* = 0.009). Overall, exposure to Me-PFOSA-AcOH was associated with CVD among non-Hispanic males with a college degree and high family income who have an abnormal BMI, smoke, engage in only moderate physical activity, and do not have hypertension. 

### 2.5. Identification of Core Targets and PPI Network Analysis

**Figure 3:**
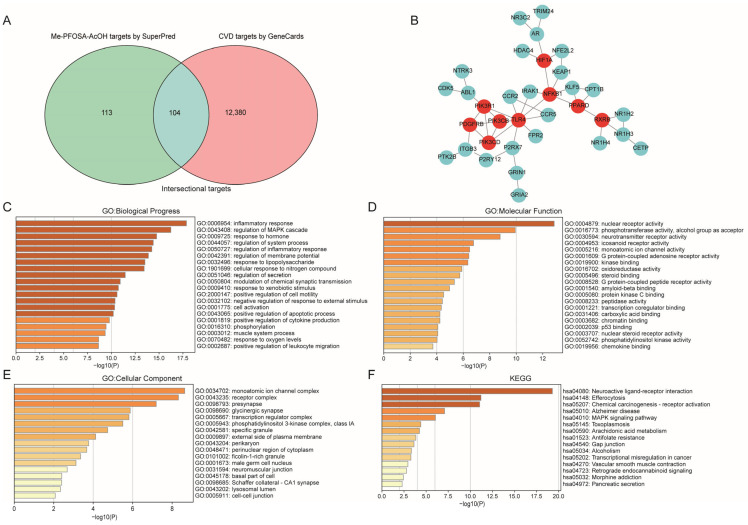
Me-PFOSA-AcOH acts on relevant targets in CVD: PPI network and bar plots of enrichment analysis. (**A**) Venn diagram indicating the intersecting genes for Me-PFOSA-AcOH and CVD. (**B**) PPI networks highlight the core targets for the action of Me-PFOSA-AcOH on CVD. Red circle indicated targets with degree value > 4 by Cytoscape 3.10.2 software. (**C**–**E**) GO functional enrichment analysis. (**F**) KEGG pathway enrichment analysis.

To delve deeply into the mechanism of Me-PFOSA-AcOH activity in the context of CVD, we conducted target gene prediction for Me-PFOSA-AcOH and CVD using SuperPred and GeneCards, respectively. A Venn diagram was generated by intersecting the 217 Me-PFOSA-AcOH targets with the 12,484 CVD targets. We identified 104 potential common targets for the impact of Me-PFOSA-AcOH on CVD (Figure 3A) and built a PPI network of these targets using STRING. The cys file was utilized in Cytoscape 3.10.2, and after eliminating isolated targets, a final PPI network with 34 nodes and 90 edges was produced (Figure 3B). We observed nine core targets with a degree value > 4 including TLR4, NFKB1, PIK3R1, HIF1A, PPARD, PDGFRB, RXRB, PIK3CD, and PIK3CB, in which the degree value of TLR4 was highest. 

### 2.6. GO and KEGG Enrichment Analyses of Intersection Targets 

GO and KEGG enrichment analysis of the 104 intersection targets were conducted using Metascape. Next, intersection targets were enriched for the following biological progress (BP) terms: inflammatory response, regulation of MAPK cascade, response to hormone, regulation of system process, and regulation of inflammatory response, among others (Figure 3C). Molecular function category (MF) enrichment terms were mainly involved in nuclear receptor activity, phosphotransferase activity, the alcohol group as an acceptor, and neurotransmitter receptor activity (Figure 3D). Additionally, enriched cellular component (CC) terms were primarily associated with the monoatomic ion channel complex and receptor complex (Figure 3E). KEGG pathway enrichment pathways were mainly involved in neuroactive ligand–receptor interaction, the MAPK signaling pathway, and arachidonic acid metabolism, among others (Figure 3F). 

### 2.7. Molecular Docking Validation of Me-PFOSA-AcOH and Core Target

**Figure 4:**
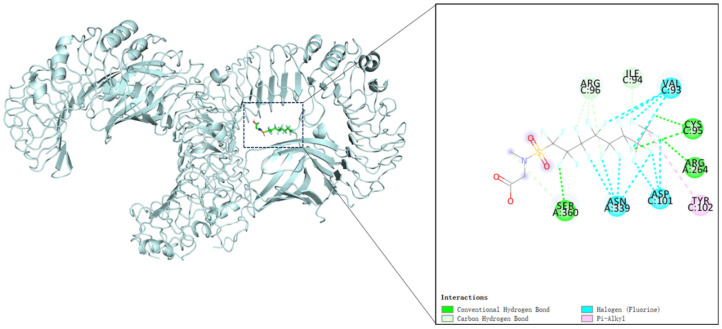
The molecular docking data of Me-PFOSA-AcOH on TLR4-MD-2 complex were analyzed using Autodock Vina 1.1.2.

To evaluate whether Me-PFOSA-AcOH can bind to target proteins identified through network analysis, we performed molecular docking of Me-PFOSA-AcOH with the key target TLR4 (degree value = 9). Molecular docking between Me-PFOSA-AcOH and the TLR4-MD-2 complex was visualized (Figure 4), and the corresponding binding energy was −7.2 kcal/mol, suggesting that Me-PFOSA-AcOH has a good binding capacity to TLR4-MD-2 complex. In detail, we found that Me-PFOSA-AcOH can form conventional hydrogen interactions with the SER360, ARG264, and CYS95 of the complex, can form carbon hydrogen with ARG96 and ILE94, can form halogen interactions with VAL93, ASN339, and ASP101, and can form pi-alkyl interactions with TYR102. In summary, we found that Me-PFOSA-AcOH binds well to TLR4-MD-2 complex, and Me-PFOSA-AcOH may lead to CVD by binding to TLR4.

## 3. Discussion 

PFASs are artificial compounds extensively utilized in both industrial and consumer products around the world; therefore, exposure to PFAS is nearly ubiquitous [32]. Although observational data suggest connections between PFAS and specific cardiac risk factors as outlined by the National Academies of Sciences, Engineering, and Medicine, further investigations are imperative to establish causative relationships and gain deeper insights into the mechanisms through which PFAS exposure contributes to the onset of these disorders [11]. In this study, we investigated several prevalent PFASs in the adult population of the US and explored their correlation with CVD and its subtypes. By employing coherent findings from multiple models, our analysis revealed that Me-PFOSA-AcOH exhibited a positive association with both CVD and its subtypes, including coronary heart disease and heart attack, even after controlling for all covariates. 

Previous research has emphasized PFAS exposure because of its connections with CVD risk factors in the general population. Reports have indicated that exposure to a mixture of PFASs elevated the risk of overall CVD, and PFOS was found to be positively associated with an increased risk of heart attack and stroke in men [33]. An observed positive correlation between rising serum PFOA levels and the risk of CVD was noted in the US population [34]. Exposure to PFAS is associated with changes in the levels of proteins that have previously been connected to cardiovascular disease in middle-aged individuals [35]. An animal study has shown that exposure to PFOS in mice results in an increased CVD risk by enhancing lipid accumulation and elevating oxidized phospholipid levels [36]. PFOSA, a precursor of PFOS, induces cardiotoxicity in zebrafish [37]. Another study demonstrated that PFOS could induced cardiac toxicity in rats by promoting cell apoptosis and the expression of proinflammatory cytokines [38]. Direct proof of a link between PFAS exposure and the prevalence of CVD is still limited, with current results being inconsistent. A meta-analysis revealed that while PFOA and PFOS were positively correlated with CVD, additional observational studies are needed to determine the long-term impact of these substances on the early onset of CVD in patients [39]. In a cohort study, no clear link was observed between PFOA exposure and the incidence of coronary artery disease [40]. Although PFAS exposure was linked to elevated cholesterol levels, it was not associated with a higher risk of myocardial infarction or stroke [26]. 

In this study, the demographic differences between the CVD and non-CVD groups highlight the importance of adjusting for potential covariates in our subsequent analyses. The increased levels of PFHxS, PFNA, n-PFOS, Sm-PFOS, and Me-PFOSA-AcOH in the CVD group suggested that these specific PFASs may be associated with CVD. This finding aligns with earlier reports linking PFAS exposure to adverse cardiovascular outcomes [20]. Then, we further observed a strong association between Me-PFOSA-AcOH and CVD, particularly coronary heart disease and heart attack in three cycles from NHANES 2013–2018. However, although PFUA showed a significant relationship with total CVD, we found no significant association between PFUA and the CVD subtypes. For n-PFOA, we found that it showed a significant relationship with total CVD in Model 3, but we found no significant association between n-PFOA and total CVD in Model 1 without adjusting any covariates, suggesting that the association was not robust. The PLS-DA model, as an artificial intelligence-based analytical approach, can be utilized for predicting toxicity and identifying toxicants [41]. Me-PFOSA-AcOH was found to be the most discriminant variable by PLS-DA, indicating its strong predictive power for CVD. In the subgroup analysis, the robust and significant positive association between Me-PFOSA-AcOH and CVD persisted primarily among male participants, non-Hispanic individuals, those with a high PIR, abnormal BMI, or those who were smokers, underscoring the consistency and strength of the observed association in specific demographic and lifestyle subgroups, reinforcing the potential role of Me-PFOSA-AcOH as a risk factor for CVD in these populations. Such findings could potentially be ascribed to the varying pharmacokinetics of Me-PFOSA-AcOH across different sexes or ethnicities [42,43]. Additionally, we found that the proportion of individuals classified as underweight (<18.5 kg/m^2^) was quite low (45/3391 = 1.3%). This small sample size may render statistical calculations infeasible due to insufficient data for estimating correlations or testing hypotheses. Therefore, in our BMI categorization, we included both underweight individuals and those with a BMI above the normal range in the abnormal BMI group. Consequently, the observed association between Me-PFOSA-AcOH and CVD in the abnormal BMI subgroup primarily pertains to individuals with a BMI above the normal range. These findings above supported the notion that Me-PFOSA-AcOH plays a significant role in the development of CVD. 

Me-PFOSA-AcOH is generated in the body through the metabolic degradation of PFOS [44]. Similar to other PFAS, it is identified as a durable and persistent substance capable of accumulating in both the environment and living organisms. It has been found in various environmental samples [45], as well as in humans [44,46]. Previous research has examined the possible health impacts of Me-PFOSA-AcOH. Me-PFOSA-AcOH exposure was negatively associated with fasting blood glucose in adolescents [47]. It is found that Me-PFOSA-AcOH was associated with later thelarche and later pubarche in young girls [48]. However, the available data in humans and animals related to the effects of Me-PFOSA-AcOH on CVD are limited, and the mechanism is still unclear. In this study, we initially unveiled the relationship between Me-PFOSA-AcOH exposure and CVD outcomes. Based on the findings in our population association study, we employed a novel analysis strategy to further elucidate its potential mechanism. 

Network toxicology analysis is a powerful approach that integrates network biology and toxicology to study the interactions between chemicals, biological targets, and diseases at a system level [49,50]. Through network toxicology analysis, we identified 104 common targets for both Me-PFOSA-AcOH and CVD. Subsequently, a PPI network was constructed to visualize interactions among these common targets in the context of Me-PFOSA-AcOH effects on CVD. TLR4, NFKB1, PIK3R1, HIF1A, PPARD, PDGFRB, RXRB, PIK3CD, and PIK3CB emerged as core targets linking Me-PFOSA-AcOH and CVD. Notably, the most key target TLR4 may serve a pivotal role in mediating the impact of Me-PFOSA-AcOH on CVD. Toll-like receptor 4 (TLR4) is an essential part of the innate immune system and is vital for detecting damage-associated molecular patterns [51]. Reports indicate that TLR4 is involved in the development of several cardiovascular diseases. Activation of TLR4 has been associated with the onset and progression of atherosclerosis [52]. Another study showed that deleting TLR4 specifically in cardiomyocytes offers protection against Ang II-induced hypertension and cardiac dysfunction by inhibiting proinflammatory cytokines [53]. TLR4 signaling can exacerbate myocardial injury and adverse remodeling in congestive heart failure [54]. TLR4 may serve as a potential clinical biomarker for cardiovascular risk in patients with coronary artery disease [55]. Following a myocardial infarction (heart attack), TLR4 signaling is involved in the tissue damage in the heart [56]. MD2 (Myeloid differentiation protein 2) is a co-receptor that forms a complex with TLR4 [57] to exert biological effects. A previous study showed that the TLR4-MD-2 complex enhances the inflammatory response [58]. Furthermore, TLR4-MD-2 complex has been reported to affect the blood pressure and vascular function [59]. In our study, the molecular docking results indicated that Me-PFOSA-AcOH bound closely to the TLR4-MD-2 complex, suggesting that Me-PFOSA-AcOH could impact the biological activity of CVD-related targets, and the reliability of the core target of Me-PFOSA-AcOH associated with CVD was further confirmed. Me-PFOSA-AcOH’s binding to the TLR4-MD-2 complex and potential modulation of TLR4 activity could disrupt immune responses and inflammatory signaling pathways, ultimately impacting cardiovascular health. Therefore, the TLR4-MD-2 complex might have a crucial role in the development of CVD induced by Me-PFOSA-AcOH. 

It has been reported that PFAS has immunotoxicity, as evaluated by the European Food Safety Authority (EFSA, https://www.efsa.europa.eu/en/efsajournal/pub/6223, accessed on 16 September 2024), and studies have shown that TLR4 mediates immunotoxicity effects [60,61]. However, we selected TLR4 as the key target related to CVD based on the following reasons: Firstly, CVD is the primary outcome of interest in our study. Secondly, we conducted a network toxicology analysis to identify core targets for both CVD and PFASs and found that TLR4 was the core target with the highest degree value (degree value = 9) related to CVD [62], making the selection of TLR4 reliable. Thirdly, previous studies have reported a close association between TLR4 and CVD [54,55,56]. Additionally, dysregulation of the immune system is closely associated with the occurrence of CVD [63,64]. Thus, TLR4 might ultimately induce CVD by disrupting the immune system. Finally, molecular docking results confirmed that Me-PFOSA-AcOH binds closely to the TLR4-MD-2 complex, which has been reported to affect blood pressure and vascular function [59]. Therefore, we consider TLR4 to be the most crucial target associated with CVD in our study. Of course, TLR4 was also linked to other disease, such as neoplastic conditions [65,66] and inflammatory ailments [67,68]. We will focus on the role of TLR4 in the development of other diseases in the future.

In addition to TLR4, other targets might also contribute to the pathogenesis of CVD induced by Me-PFOSA-AcOH. NFKB1 is a critical transcription factor that regulates inflammatory responses, with mutations linked to the occurrence of significant adverse cardiovascular events [69]. PI3K/Akt signaling, in which PIK3R1 plays a regulatory role, is a crucial pathway involved in cell survival, proliferation, and metabolism. Modulation of PI3K/Akt signaling may impact cardiac function and vascular health [70]. In cardiovascular diseases, HIF1A is involved in the adaptation of the heart and blood vessels to low oxygen levels [71]. PPARD and RXRB are involved in lipid metabolism and inflammation, whose dysregulation might impact cardiovascular health [72]. Aneurysms are vascular abnormalities that have been documented in various patients with PDGFRB-related conditions [73]. PIK3CD and PIK3CB are catalytic subunits of phosphoinositide-3-kinase (PI3K) involved in intracellular signaling pathways regulating cell growth, proliferation, and survival. Modulation of PI3K activity may have implications for cardiovascular health and disease progression [74]. GO and KEGG enrichment analyses of the key targets further supported the involvement of inflammatory responses, the MAPK signaling pathway, and lipid metabolism in the context of Me-PFOSA-AcOH and CVD. These results offer evidence of the biological significance of Me-PFOSA-AcOH in the progression of CVD. 

In comparison to previous reports, this study possesses some advantages. First, our study included a relatively large sample size, allowing for robust statistical analyses and subgroup analyses. Second, we examined multiple PFASs, providing a comprehensive assessment of their associations with CVD. Third, we first conducted target gene prediction, constructed a PPI network, and performed molecular docking, shedding light on the molecular mechanisms underlying the impact of Me-PFOSA-AcOH on CVD.

Despite these strengths, our study has the following limitations. First, the cross-sectional design restricts our capacity to determine causality between Me-PFOSA-AcOH and CVD. Second, we relied on self-reported data for some variables, which may introduce recall bias. Third, the low levels of Me-PFOA-AcOH may impact the robustness of our findings, which emphasize the need for further validation through independent studies. Finally, our study lacks in vivo and in vitro studies to validate the core targets revealed by molecular docking. Longitudinal studies and in vivo and in vitro models are needed to further confirm the causal relationship. 

## 4. Conclusions 

In conclusion, our research demonstrates a connection between Me-PFOSA-AcOH and CVD, especially coronary heart disease and heart attacks. TLR4, a novel molecular target revealed by molecular docking and network toxicology, has been shown to be involved in the effects of Me-PFOSA-AcOH on CVD. Our results offer new perspectives on the molecular targets and mechanisms involved in Me-PFOSA-AcOH-induced CVD. These results are significant for public health and emphasize the need for additional research and regulatory measures to lower PFAS exposure and reduce CVD risk. 

## 5. Methods

### 5.1. Study Population 

**Figure 5:**
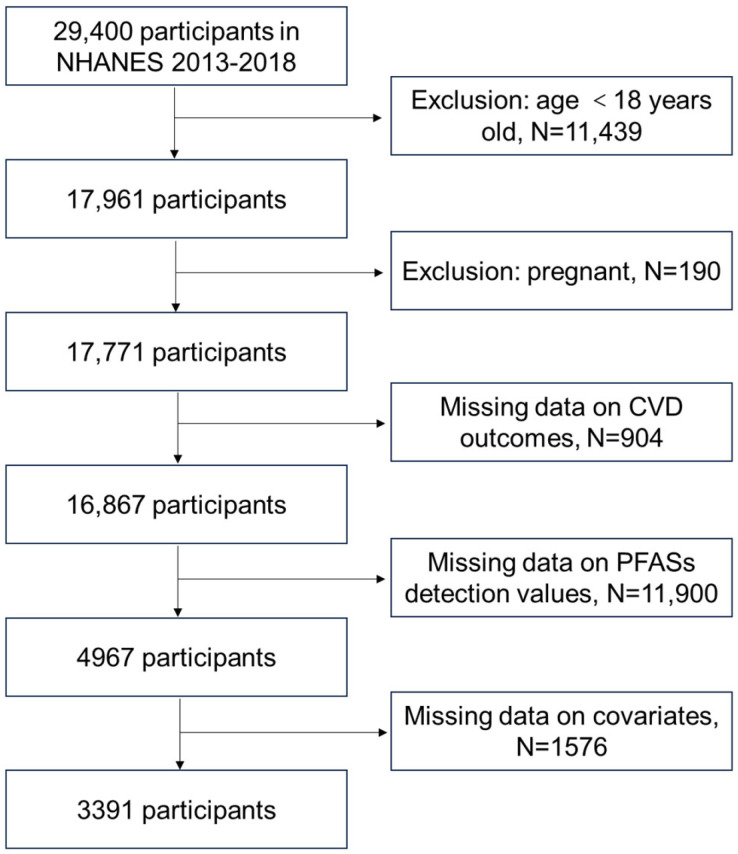
Selection of study participants.

NHANES aims to evaluate the health and dietary condition of both adults and children in the United States by means of interviews, physical assessments, and laboratory analyses [75]. In this study, data from 29,400 participants were included from 3 cycles of NHANES (2013–2014, 2015–2016, 2017–2018). The criteria for exclusion were as follows: (a) participants under 18 years old, totaling 11439 individuals; (b) pregnant individuals, with 190 excluded; (c) those with incomplete data for CVD outcome indicators, totaling 904 exclusions; (d) individuals lacking PFAS detection values, with 11,900 excluded; (e) participants with missing covariate values, totaling 1576 exclusions. Consequently, 3391 adults were retained for the final analysis. The exclusion procedure is depicted in Figure 5. The study received approval from the National Center for Health Statistics Research Ethics Review Board, and all participants provided written informed consent. 

### 5.2. Ascertainment of CVD

In this study, CVD was defined according to a previous study [17]. In brief, we included five variables from NHANES related to CVD outcomes: “MCQ160B”, “MCQ160C”, “MCQ160D”, “MCQ160E”, and “MCQ160F”. All participants were questioned concerning the following subjects: “Has a doctor or other health professional ever told you that you had congestive heart failure (CHF)?/coronary heart disease (CHD)?/angina pectoris?/heart attack?/stroke?”. If participants answered “yes” to any of the aforementioned five questions, they were categorized as patients with CVD. If participants did not answer “yes” to any of the questions and there were no missing data for all questions, they were classified as non-CVD. Participants with missing data for all the five questions were removed. 

### 5.3. PFASs Measurements

The analysis of PFASs in blood serum was carried out according to a previous study [76]. In brief, serum PFASs were identified through using online solid phase extraction combined with high performance liquid chromatography–turbo ion spray ionization–tandem mass spectrometry (online SPE-HPLC-TIS-MS/MS). If the concentration of any analyte is less than the limit of detection (LOD), the value is substituted with the LOD divided by the square root of two [77]. PFASs analyzed in our study were detected in more than 65% of the included participants, including perfluorodecanoic acid (PFDeA), perfluorohexane sulfonic acid (PFHxS), 2-(N-methylperfluoroctanesulfonamido)acetic acid (Me-PFOSA-AcOH), perfluorononanoic acid (PFNA), perfluoroundecanoic acid (PFUA), n-perfluorooctanoic acid (n-PFOA), n-perfluorooctane sulfonic acid (n-PFOS), and perfluoromethylheptane sulfonic acid isomers (Sm-PFOS). The raw data of PFASs in blood serum were from NHANES 2013–2018. 

### 5.4. Covariates

Sex, age, ethnicity, education level, family income-to-poverty ratio (PIR) [78], physical activity, smoking status, drinking status, family history of CVD, body mass index (BMI), and hypertension were included as potential covariates in our study. Smoking status was classified based on whether individuals had consumed at least 100 cigarettes over their lifetime or not. Drinking status was divided into 4 groups: never drinkers (0 drinks per week), light drinkers (less than 1 drink per week), moderate drinkers (between 1 and 8 drinks per week), and heavy drinkers (8 or more drinks per week) [79]. Family history of CVD was classified based on whether or not individuals had close blood relatives who experienced a heart attack or angina before the age of 50. BMI was classified into categories as follows: underweight (BMI < 18.5 kg/m^2^), normal weight (18.5 kg/m^2^ ≤ BMI < 25 kg/m^2^), overweight (25 kg/m^2^ ≤ BMI < 30 kg/m^2^), and obese (BMI ≥ 30 kg/m^2^) [80]. Hypertension was defined as individuals with a systolic blood pressure of 130 mm Hg or higher, or a diastolic blood pressure of 80 mm Hg or greater [81]. Information regarding the measurement methods for these variables can be found on the official CDC website at https://www.cdc.gov/nchs/nhanes/ (accessed on 16 April 2024). 

### 5.5. Me-PFOSA-AcOH and CVD Targets Prediction

To gather information on the characterization and physicochemical properties of Me-PFOSA-AcOH, the PubChem database (https://pubchem.ncbi.nlm.nih.gov/, accessed on 16 April 2024) was queried using the search term CAS: 2355-31-9. Target prediction was performed with SuperPred (https://prediction.charite.de/subpages/target_prediction.php, accessed on 16 April 2024), employing Canonical SMILES for the prediction process. The predicted targets were then mapped to gene names using bioDBnet (https://biodbnet-abcc.ncifcrf.gov/db/db2db.php, accessed on 16 April 2024). The resulting gene targets were identified as those associated with Me-PFOSA-AcOH. Additionally, the GeneCards database (https://www.genecards.org/, accessed on 16 April 2024) was used to identify CVD targets. 

### 5.6. Construction of Me-PFOSA-AcOH and CVD Intersection Targets Network

We utilized the STRING database (https://cn.string-db.org/, accessed on 16 April 2024) to acquire protein–protein interaction (PPI) networks for the identified shared targets. The downloaded tsv file was then imported into Cytoscape 3.10.2 software for visualization. 

### 5.7. GO and KEGG Enrichment Analyses 

GO enrichment and KEGG pathway analyses of the target genes were conducted using Metascape (https://metascape.org/gp/index.html, accessed on 16 April 2024). The enrichment parameters for pathways and processes were configured as follows: minimum overlap of 3, *p* value cutoff of 0.01, and minimum enrichment of 1.5. Terms with a *p* value < 0.01 were considered significantly enriched in GO terms or KEGG pathways. Enriched terms were chosen based on the cluster results from Metascape.

### 5.8. Molecular Docking

We retrieved the SDF format file for Me-PFOSA-AcOH from PubChem (https://pubchem.ncbi.nlm.nih.gov/, accessed on 16 April 2024) and obtained the PDB format file for the human TLR4-MD-2 complex (PDB code: 3FXI) from the PDB database (https://www.rcsb.org/, accessed on 16 April 2024). Molecular docking was conducted using Autodock Vina 1.1.2, with the binding potential of Me-PFOSA-AcOH to the protein evaluated based on the binding energy values. The outcomes of the molecular docking were visualized using PyMOL (version 1.2) and Discovery Studio (version 2.4). 

### 5.9. Statistical Analysis

For continuous variables adhering to a normal distribution, mean values with standard deviation (SD) are presented, while for those not following a normal distribution, median values (first quartile (Q1), third quartile (Q3)) are provided. Categorical variables are described using percentages. The Kolmogorov–Smirnov test was employed to evaluate the normal distribution and variance homogeneity for all data. A rank sum test was used to compare the non-CVD group with the CVD group, while the chi-square test was applied to categorical variables. To address the skewed distributions, serum PFAS levels were transformed using the natural logarithm (ln-PFAS). Following this, potential associations between each PFAS and the risk of overall CVD or its subtypes (CHF, CHD, angina pectoris, heart attack, and stroke) were examined using multivariable logistic regression models. To adjust for covariates in a stepwise manner, three models were constructed. Model 1 did not include any adjustments. Model 2 was adjusted for demographic factors, such as sex, age, and ethnicity. Model 3 was adjusted for the same demographic variables as Model 2 and additionally accounted for education level, PIR, physical activity, smoking status, drinking status, family history of CVD, BMI, and hypertension. To investigate whether the associations were modified by covariates, subgroup analyses were performed. PLS-DA was employed to identify the PFAS which contributed the most to distinguishing between CVD and non-CVD cases. The data analysis was performed using R version 4.2.0. The threshold for statistical significance was established at *p* < 0.05. Due to the complex and multi-stage probability survey design, weights were applied where appropriate in the analyses in this study in accordance with the NHANES analysis guidelines. 

## Figures and Tables

**Table 1:** Characteristics of the study participants (n = 3391), NHANES 2013–2018 ^a^.

Characteristic (%)	Non-CVD	CVD	*p* Value
	(n = 3020)	(n = 371)	
Sex			0.257
Male	50.3	54.8	
Female	49.7	45.2	
Age			<0.001 *
<60 years old	76.5	30.4	
≥60 years old	23.5	69.6	
Ethnicity			0.001 *
Non-Hispanic	85.4	92.0	
Hispanic	14.6	8.0	
Education level			0.006 *
Less than high school	11.0	16.8	
High school graduate	22.9	30.4	
Some college or AA degree	32.9	30.1	
College graduate or above	33.2	22.7	
PIR			0.020 *
≤1	12.0	17.2	
1–3	34.0	38.1	
>3	54.0	44.7	
BMI			0.005 *
Underweight	1.4	0.8	
Normal weight	26.9	19.1	
Overweight	31.7	32.6	
Obesity	40.1	47.4	
Smoking status			<0.001 *
No	56.2	31.6	
Yes	43.8	68.4	
Drinking status			<0.001 *
Never drinkers	14.0	29.9	
Light drinkers	36.7	40.1	
Moderate drinkers	33.0	24.3	
Heavy drinkers	16.3	5.7	
Physical activity			<0.001 *
Never	42.1	60.0	
Moderate	28.6	30.5	
Vigorous	29.3	9.5	
Family history of CVD			<0.001 *
No	87.0	73.4	
Yes	13.0	26.6	
Hypertension			<0.001 *
No	65.2	50.5	
Yes	34.8	49.5	

^a^ Categorical variables were listed as weighted percentage. * *p* < 0.05.

**Table 2:** Distribution of blood serum PFASs concentrations in the participants, weighted.

PFAS Median (Q1, Q3)	Non-CVD (n = 3020)	CVD (n = 371)	*p* Value
PFDeA (ng/mL)	0.20 (0.10, 0.30)	0.20 (0.10, 0.30)	0.038 *
PFHxS (ng/mL)	1.30 (0.70, 2.20)	1.60 (0.90, 2.80)	0.010 *
PFNA (ng/mL)	0.60 (0.40, 0.90)	0.70 (0.40, 1.10)	0.025 *
PFUA (ng/mL)	0.10 (0.07, 0.20)	0.10 (0.07, 0.20)	0.028 *
n-PFOA (ng/mL)	1.50 (1.00, 2.20)	1.60 (1.00, 2.20)	0.878
n-PFOS (ng/mL)	3.30 (2.00, 5.80)	4.80 (2.80, 8.50)	0.003 *
Sm-PFOS (ng/mL)	1.50 (0.80, 2.60)	2.10 (1.10, 3.70)	0.030 *
Me-PFOSA-AcOH (ng/mL)	0.07 (0.07, 0.20)	0.10 (0.07, 0.30)	<0.001 *

* *p* < 0.05.

**Table 3:** Logistic regression analysis of the association between the blood serum PFAS levels and CVD in the participants, weighted.

PFAS	Model 1		Model 2		Model 3	
	OR (95% CI)	*p* Value	OR (95% CI)	*p* Value	OR (95% CI)	*p* Value
PFDeA	1.16 (1.00, 1.35)	0.061	0.92 (0.78, 1.08)	0.300	1.12 (0.92, 1.36)	0.272
PFHxS	1.23 (0.99, 1.53)	0.074	0.88 (0.68, 1.14)	0.349	0.93 (0.73, 1.19)	0.587
PFNA	1.19 (0.98, 1.44)	0.089	0.86 (0.70, 1.04)	0.126	0.95 (0.76, 1.19)	0.674
PFUA	1.30 (1.09, 1.55)	0.006 *	1.04 (0.87, 1.26)	0.658	1.32 (1.08, 1.61)	0.012 *
n-PFOA	0.87 (0.65, 1.16)	0.360	0.65 (0.49, 0.85)	0.005 *	0.73 (0.56, 0.96)	0.035 *
n-PFOS	1.47 (1.04, 2.09)	0.039 *	0.97 (0.68, 1.39)	0.875	1.06 (0.74, 1.53)	0.751
Sm-PFOS	1.31 (0.95, 1.79)	0.109	0.81 (0.59, 1.11)	0.206	0.89 (0.64, 1.22)	0.468
Me-PFOSA-AcOH	1.58 (1.35, 1.85)	<0.001 *	1.34 (1.10, 1.64)	0.006 *	1.28 (1.05, 1.56)	0.022 *

Model 1: adjusted for no covariates. Model 2: adjusted for sex, age, and ethnicity. Model 3: adjusted for sex, age, ethnicity, education level, PIR, physical activity, smoking status, drinking status, family history of CVD, BMI, and hypertension. * *p* < 0.05.

## Data Availability

Publicly available datasets were analyzed in this study. The data can be found at https://wwwn.cdc.gov/nchs/nhanes/Default.aspx (accessed on 16 April 2024).

## Supplementary Materials

The following supporting information can be downloaded at: https://www.mdpi.com/article/10.3390/toxics12100693/s1, Table S1: Logistic regression analysis for the association between blood serum PFAS levels and CVD subtypes in the participants, weighted.
