# Supplementary material for: TLR4 as a Potential Target of Me-PFOSA-AcOH Leading to Cardiovascular Diseases: Evidence from NHANES 2013–2018 and Molecular Docking
Source: Toxics. 2024 Sep 25;12(10):693. doi: 10.3390/toxics12100693 (PMC11511422; doi:10.3390/toxics12100693)
Supplement: Supplementary file 1 [file toxics-12-00693-s001.zip › toxics-3174105-supplementary.pdf]

Supplemental materials

Table S1. Logistic regression analysis for the association between serum PFAS levels and CVD subtypes in the participants, weighted.

CVD subtype	PFAS	Model 1		Model 2		Model 3	
		OR (95%CI)	P value	OR (95%CI)	P value	OR (95%CI)	P value
congestive heart failure	PFDeA	0.90 (0.62, 1.33)	0.613	0.72 (0.49, 1.07)	0.111	0.88 (0.58, 1.33)	0.549
	PFHxS	0.86 (0.66, 1.11)	0.254	0.62 (0.48, 0.81)	0.001*	0.64 (0.48, 0.85)	0.004*
	PFNA	0.91 (0.67, 1.23)	0.525	0.69 (0.53, 0.91)	0.011*	0.74 (0.54, 1.02)	0.072
	PFUA	1.01 (0.70, 1.46)	0.938	0.82 (0.56, 1.21)	0.327	1.06 (0.69, 1.63)	0.786
	n-PFOA	0.76 (0.54, 1.06)	0.360	0.60 (0.46, 0.80)	0.005*	0.72 (0.51, 1.01)	0.035*
	n-PFOS	0.91 (0.58, 1.43)	0.039*	0.62 (0.43, 0.87)	0.875	0.67 (0.46, 0.96)	0.751
	Sm-PFOS	0.93 (0.65, 1.32)	0.109	0.60 (0.44, 0.81)	0.206	0.65 (0.47, 0.90)	0.468
	Me-PFOSA-AcOH	1.54 (1.23, 1.92)	0.000*	1.33 (1.05, 1.68)	0.024*	1.24 (0.95, 1.61)	0.122
coronary heart disease	PFDeA	1.17 (0.91, 1.51)	0.238	0.85 (0.65, 1.13)	0.270	0.96 (0.71, 1.31)	0.800
	PFHxS	1.39 (1.00, 1.93)	0.059	0.89 (0.61, 1.30)	0.559	0.92 (0.62, 1.37)	0.680
	PFNA	1.43 (1.05, 1.96)	0.030*	0.94 (0.69, 1.27)	0.683	1.01 (0.71, 1.42)	0.969
	PFUA	1.32 (0.99, 1.75)	0.063	0.99 (0.73, 1.34)	0.932	1.14 (0.82, 1.56)	0.441
	n-PFOA	0.91 (0.51, 1.63)	0.360	0.66 (0.41, 1.06)	0.005*	0.68 (0.40, 1.16)	0.035*
	n-PFOS	1.71 (1.02, 2.86)	0.039*	1.01 (0.63, 1.61)	0.875	1.03 (0.61, 1.73)	0.751
	Sm-PFOS	1.56 (0.90, 2.73)	0.109	0.87 (0.54, 1.38)	0.206	0.91 (0.55, 1.49)	0.468
	Me-PFOSA-AcOH	1.80 (1.47, 2.20)	0.000*	1.50 (1.16, 1.94)	0.004*	1.47 (1.13, 1.92)	0.007*
angina pectoris	PFDeA	1.04 (0.77, 1.41)	0.807	0.85 (0.61, 1.17)	0.325	1.08 (0.77, 1.50)	0.668
	PFHxS	0.89 (0.58, 1.38)	0.605	0.64 (0.44, 0.95)	0.032*	0.72 (0.50, 1.03)	0.085
	PFNA	1.13 (0.75, 1.72)	0.563	0.86 (0.60, 1.24)	0.437	1.00 (0.67, 1.49)	0.985
	PFUA	1.20 (0.92, 1.57)	0.194	0.99 (0.74, 1.32)	0.961	1.28 (0.99, 1.66)	0.072

	n-PFOA	0.76 (0.37, 1.56)	0.360	0.64 (0.38, 1.08)	0.005*	0.76 (0.41, 1.38)	0.035*
	n-PFOS	1.18 (0.61, 2.29)	0.039*	0.87 (0.51, 1.50)	0.875	1.01 (0.59, 1.73)	0.751
	Sm-PFOS	1.13 (0.56, 2.28)	0.109	0.80 (0.47, 1.38)	0.206	0.91 (0.53, 1.56)	0.468
	Me-PFOSA-AcOH	1.50 (1.13, 2.00)	0.007*	1.30 (0.95, 1.78)	0.106	1.22 (0.91, 1.64)	0.195
heart attack	PFDeA	1.13 (0.87, 1.48)	0.362	0.89 (0.67, 1.18)	0.421	1.06 (0.78, 1.43)	0.727
	PFHxS	1.34 (1.09, 1.65)	0.009*	0.96 (0.70, 1.30)	0.785	1.00 (0.74, 1.33)	0.974
	PFNA	1.33 (0.99, 1.77)	0.061	0.96 (0.72, 1.27)	0.755	1.07 (0.77, 1.48)	0.695
	PFUA	1.23 (0.87, 1.74)	0.244	0.98 (0.68, 1.40)	0.910	1.18 (0.83, 1.68)	0.372
	n-PFOA	0.82 (0.66, 1.03)	0.360	0.64 (0.49, 0.83)	0.005*	0.72 (0.52, 0.98)	0.035*
	n-PFOS	1.68 (1.21, 2.33)	0.039*	1.12 (0.77, 1.63)	0.875	1.17 (0.79, 1.74)	0.751
	Sm-PFOS	1.44 (1.05, 1.98)	0.109	0.91 (0.65, 1.26)	0.206	1.00 (0.70, 1.43)	0.468
	Me-PFOSA-AcOH	1.89 (1.58, 2.26)	0.000*	1.65 (1.30, 2.09)	0.000*	1.58 (1.26, 1.99)	0.000*
stroke	PFDeA	1.06 (0.82, 1.36)	0.665	0.86 (0.66, 1.13)	0.291	1.08 (0.84, 1.39)	0.551
	PFHxS	1.17 (0.75, 1.81)	0.497	0.95 (0.58, 1.56)	0.837	1.00 (0.64, 1.57)	0.992
	PFNA	1.08 (0.78, 1.49)	0.659	0.84 (0.63, 1.12)	0.245	0.96 (0.69, 1.33)	0.796
	PFUA	1.25 (0.99, 1.58)	0.063	1.04 (0.81, 1.33)	0.786	1.28 (1.04, 1.58)	0.024*
	n-PFOA	0.81 (0.54, 1.20)	0.360	0.67 (0.49, 0.92)	0.005*	0.76 (0.54, 1.05)	0.035*
	n-PFOS	1.30 (0.80, 2.11)	0.039*	0.95 (0.59, 1.53)	0.875	1.03 (0.65, 1.63)	0.751
	Sm-PFOS	1.13 (0.73, 1.74)	0.109	0.79 (0.54, 1.16)	0.206	0.85 (0.57, 1.26)	0.468
	Me-PFOSA-AcOH	1.48 (1.16, 1.88)	0.002*	1.24 (0.94, 1.64)	0.137	1.16 (0.88, 1.52)	0.302

Model1: adjusted for no covariates.

Model2: adjusted for sex, age, and ethnicity.

Model3: adjusted for sex, age, ethnicity, education level, PIR, physical activity, smoking status, drinking status, family history of CVD, BMI, and hypertension.

* $P < 0.05$.
